# 2-(9,9-Diethyl-9*H*-fluoren-2-yl)-1-benzofuran

**DOI:** 10.1107/S1600536811006957

**Published:** 2011-02-26

**Authors:** Ping-Hsin Huang, Kai-Ling Lin, Yuh-Sheng Wen

**Affiliations:** a Cardinal Tien College of Healthcare & Management, Taipei, Taiwan 231; b Institute of Chemistry, Academia Sinica, Nankang, Taipei, Taiwan

## Abstract

In the title compound, C_25_H_22_O, the dihedral angle between the benzofuran and fluorene ring systems is 9.06 (6)°, and that between the two benzene rings of the fluorene system is 1.78 (12)°. Weak inter­molecular C—H⋯π inter­actions help to stabilize the crystal structure.

## Related literature

The title compound is a precursor for the production of hole-transporting and/or emitting materials, see: Shen *et al.* (2005 ▶). For a related structure, see: Bak *et al.* (1961 ▶).
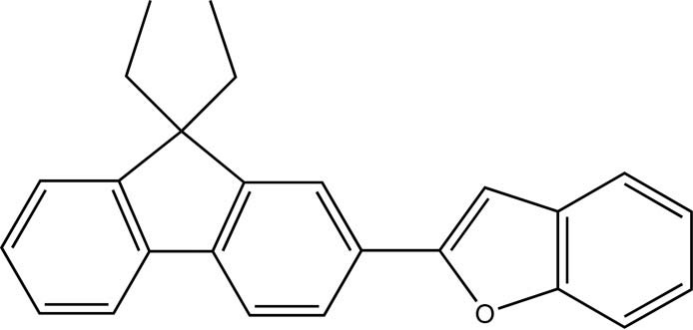


## Experimental

### 

#### Crystal data

C_25_H_22_O
*M* *_r_* = 338.43
Orthorhombic, 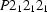

*a* = 7.5277 (13) Å
*b* = 12.9969 (19) Å
*c* = 18.438 (3) Å
*V* = 1803.9 (5) Å^3^
*Z* = 4
Mo *K*α radiation
μ = 0.07 mm^−1^
*T* = 100 K
0.2 × 0.14 × 0.08 mm

#### Data collection

Bruker SMART CCD area-detector diffractometer
8855 measured reflections
2118 independent reflections
1518 reflections with *I* > 2σ(*I*)
*R*_int_ = 0.103

#### Refinement

*R*[*F* ^2^ > 2σ(*F* ^2^)] = 0.058
*wR*(*F* ^2^) = 0.146
*S* = 1.03
2118 reflections
236 parameters
H-atom parameters constrained
Δρ_max_ = 0.23 e Å^−3^
Δρ_min_ = −0.26 e Å^−3^

### 

Data collection: *SMART* (Bruker, 2001 ▶); cell refinement: *SAINT* (Bruker, 2001 ▶); data reduction: *SAINT*; program(s) used to solve structure: *SHELXS97* (Sheldrick, 2008 ▶); program(s) used to refine structure: *SHELXL97* (Sheldrick, 2008 ▶); molecular graphics: *ORTEP-3 for Windows* (Farrugia, 1997 ▶); software used to prepare material for publication: *WinGX* (Farrugia, 1999 ▶).

## Supplementary Material

Crystal structure: contains datablocks global, I. DOI: 10.1107/S1600536811006957/rn2074sup1.cif

Structure factors: contains datablocks I. DOI: 10.1107/S1600536811006957/rn2074Isup2.hkl

Additional supplementary materials:  crystallographic information; 3D view; checkCIF report

## Figures and Tables

**Table 1:** Hydrogen-bond geometry (Å, °) *Cg*1, *Cg*2, *Cg*3 and *Cg*4 are the centroids of the C23–C28, O29/C21–C23/C28 and C2–C5/C11/C12 rings, respectively.

*D*—H⋯*A*	*D*—H	H⋯*A*	*D*⋯*A*	*D*—H⋯*A*
C2—H2⋯*Cg*1^i^	0.95	2.99	3.643 (5)	127
C22—H22⋯*Cg*1^ii^	0.95	2.70	3.500 (4)	143
C24—H24⋯*Cg*2^ii^	0.95	2.85	3.569 (5)	133
C27—H27⋯*Cg*2^iii^	0.95	2.75	3.558 (5)	143

Symmetry codes: (i) 
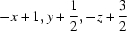
; (ii) 
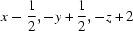
; (iii) 
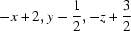
.

**Fig. 1.**
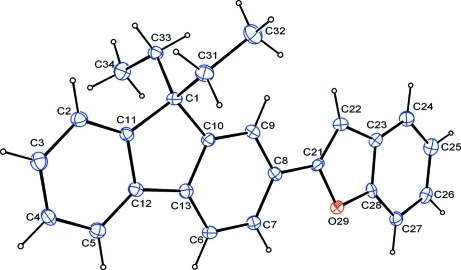
A molecular structure of (I) with 30% probability displacement ellipsoids, showing the atom-numbering scheme employed. H atoms are shown as small spheres of the arbitrary radii.

## supplementary crystallographic information

### Comment

The title compound, (I), has been shown to be an precursor for the production of hole transporting and/or emitting materials (Shen *et al.*, 2005). A one pot synthesis of a benzofuran substituted in the 2-position has been achieved by Pd(0) complex catalyzed Sonogashira coupling reaction of 2-iodophenol with terminal alkynes, followed by cyclization of the internal alkynes formed, in high yield (see scheme 1). The molecular structure is shown in Fig. 1. The dihedral angle between the benzofuran and fluorene rings is 9.06 (6)°, and that between the two benzene rings at fluorene is 1.78 (12)°. Weak intermolecular C—H···π interactions help to stabilize the crystal structure.

### Experimental

The compound was synthesized by the following procedure. A two-necked round-bottomed flask was charged with PdCl_2_(PPh_3_)_2_ (100 mg), 9,9-diethyl-2-ethynyl-9*H*-fluorene (1.35 g, 5.46 mmol), CuI (30 mg), 2-iodophenol (1.00 g, 4.55 mmol), triethylamine (1.3 ml), and DMF (10 ml), and the reaction mixture stirred under nitrogen and heated at 333 K for 24 h. After cooling, the mixture was diluted with diethyl ether and the organic phase was washed with water and brine. After drying over anhydrous MgSO_4_ and removing the volatiles, the residue was purified by column chromatography using CH_2_Cl_2_/n-hexane as eluent, followed by recrystallization from CH_2_Cl_2_ and hexane to yield 0.9 g (59%) of (I) as a white solid. Crystals suitable for X-ray diffraction were grown from a CH_2_Cl_2_ solution layered with hexane at room temperature. ^1^H NMR (CDCl_3_): 7.84 (d, 2 H, J = 7.97 Hz), 7.73 (dd, 2 H, J = 7.77 Hz), 7.55 (dd, 2 H, J = 7.64 Hz), 7.35–7.31 (m, 3 H), 7.24 (tt, 2 H, J = 8.31 Hz), 7.06 (s, 1 H), 2.09 (q, 4 H, J = 7.07 Hz), 0.34 (t, 6 H, J = 6.72 Hz). FAB MS (m/e): 338.1 (*M*^+^) Anal. Calcd for C_25_H_22_O: C, 88.72; H, 6.55. Found: C, 88.92; H, 6.51.

### Refinement

H atoms were located geometrically and treated as riding atoms, with C—H = 0.93 Å, and with *U*_iso_(H) = 1.2*U*_eq_(C). In the absence of significant anomalous scattering effects Friedel pairs have been merged.

### Crystal data

**Table:**

C_25_H_22_O	*F*(000) = 720
*M**_r_* = 338.43	*D*_x_ = 1.246 Mg m^−^^3^
Orthorhombic, *P*2_1_2_1_2_1_	Mo *K*α radiation, λ = 0.71073 Å
Hall symbol: P 2ac 2ab	Cell parameters from 525 reflections
*a* = 7.5277 (13) Å	θ = 2.7–20.4°
*b* = 12.9969 (19) Å	µ = 0.07 mm^−^^1^
*c* = 18.438 (3) Å	*T* = 100 K
*V* = 1803.9 (5) Å^3^	Prism, colourless
*Z* = 4	0.2 × 0.14 × 0.08 mm

### Data collection

**Table:**

Bruker SMART CCD area-detector diffractometer	1518 reflections with *I* > 2σ(*I*)
Radiation source: fine-focus sealed tube	*R*_int_ = 0.103
graphite	θ_max_ = 26.4°, θ_min_ = 1.9°
ω and φ scans	*h* = −8→9
8855 measured reflections	*k* = −16→16
2118 independent reflections	*l* = −17→23

### Refinement

**Table:**

Refinement on *F*^2^	Secondary atom site location: difference Fourier map
Least-squares matrix: full	Hydrogen site location: inferred from neighbouring sites
*R*[*F*^2^ > 2σ(*F*^2^)] = 0.058	H-atom parameters constrained
*wR*(*F*^2^) = 0.146	*w* = 1/[σ^2^(*F*_o_^2^) + (0.*P*)^2^] where *P* = (*F*_o_^2^ + 2*F*_c_^2^)/3
*S* = 1.03	(Δ/σ)_max_ < 0.001
2118 reflections	Δρ_max_ = 0.23 e Å^−^^3^
236 parameters	Δρ_min_ = −0.26 e Å^−^^3^
0 restraints	Extinction correction: *SHELXL97* (Sheldrick, 2008), Fc^*^=kFc[1+0.001xFc^2^λ^3^/sin(2θ)]^-1/4^
Primary atom site location: structure-invariant direct methods	Extinction coefficient: 0.007 (2)

### Special details

**Table:**

Experimental. ^1^H NMR (CDCl_3_): 7.77 (d, J = 8.2, 4H), 7.64 (d, J = 8.2, 4H). FAB MS (m/e): 462 (*M*^+^). Analysis calculated for C_18_H_8_F_6_N_2_O_4_S: C 46.76, H 1.74, N 6.06%; found: C 46.80, H 1.88, N 5.79%.
Geometry. All e.s.d.'s (except the e.s.d. in the dihedral angle between two l.s. planes) are estimated using the full covariance matrix. The cell e.s.d.'s are taken into account individually in the estimation of e.s.d.'s in distances, angles and torsion angles; correlations between e.s.d.'s in cell parameters are only used when they are defined by crystal symmetry. An approximate (isotropic) treatment of cell e.s.d.'s is used for estimating e.s.d.'s involving l.s. planes.
Refinement. Refinement of *F*^2^ against ALL reflections. The weighted *R*-factor *wR* and goodness of fit *S* are based on *F*^2^, conventional *R*-factors *R* are based on *F*, with *F* set to zero for negative *F*^2^. The threshold expression of *F*^2^ > σ(*F*^2^) is used only for calculating *R*-factors(gt) *etc*. and is not relevant to the choice of reflections for refinement. *R*-factors based on *F*^2^ are statistically about twice as large as those based on *F*, and *R*- factors based on ALL data will be even larger.

### Fractional atomic coordinates and isotropic or equivalent isotropic displacement parameters (Å^2^)

**Table:**

	*x*	*y*	*z*	*U*_iso_*/*U*_eq_	
O29	0.8727 (4)	0.12706 (16)	0.89298 (15)	0.0233 (6)	
C1	0.6113 (5)	0.4536 (2)	0.6667 (2)	0.0210 (9)	
C2	0.5957 (6)	0.4834 (3)	0.5267 (2)	0.0241 (9)	
H2	0.5355	0.5473	0.5313	0.029*	
C3	0.6407 (6)	0.4451 (3)	0.4588 (2)	0.0274 (10)	
H3	0.6092	0.4828	0.4166	0.033*	
C4	0.7307 (6)	0.3529 (2)	0.4517 (2)	0.0248 (9)	
H4	0.7610	0.3284	0.4048	0.030*	
C5	0.7768 (6)	0.2960 (2)	0.5121 (2)	0.0250 (9)	
H5	0.8382	0.2325	0.5070	0.030*	
C6	0.8406 (5)	0.2003 (2)	0.6748 (2)	0.0234 (9)	
H6	0.8885	0.1541	0.6400	0.028*	
C7	0.8484 (5)	0.1773 (3)	0.7477 (2)	0.0246 (9)	
H7	0.9038	0.1152	0.7628	0.030*	
C8	0.7765 (5)	0.2434 (2)	0.7995 (2)	0.0203 (8)	
C9	0.6972 (5)	0.3362 (2)	0.7766 (2)	0.0221 (9)	
H9	0.6485	0.3823	0.8113	0.027*	
C10	0.6904 (5)	0.3599 (2)	0.7040 (2)	0.0204 (9)	
C11	0.6400 (5)	0.4271 (2)	0.5872 (2)	0.0197 (9)	
C12	0.7322 (5)	0.3329 (2)	0.5805 (2)	0.0210 (8)	
C13	0.7618 (5)	0.2920 (2)	0.6527 (2)	0.0211 (8)	
C21	0.7791 (5)	0.2166 (2)	0.8762 (2)	0.0218 (9)	
C22	0.7057 (6)	0.2573 (2)	0.9356 (2)	0.0236 (9)	
H22	0.6381	0.3189	0.9376	0.028*	
C23	0.7468 (6)	0.1913 (2)	0.9960 (2)	0.0238 (9)	
C24	0.7066 (6)	0.1876 (3)	1.0693 (2)	0.0293 (9)	
H24	0.6388	0.2407	1.0914	0.035*	
C25	0.7675 (6)	0.1048 (3)	1.1097 (2)	0.0311 (10)	
H25	0.7404	0.1012	1.1600	0.037*	
C26	0.8683 (6)	0.0264 (3)	1.0779 (2)	0.0285 (10)	
H26	0.9081	−0.0295	1.1069	0.034*	
C27	0.9113 (6)	0.0287 (3)	1.0048 (2)	0.0251 (9)	
H27	0.9802	−0.0238	0.9826	0.030*	
C28	0.8480 (5)	0.1119 (2)	0.9661 (2)	0.0203 (9)	
C31	0.7154 (6)	0.5539 (2)	0.6825 (2)	0.0283 (10)	
H31A	0.8425	0.5409	0.6722	0.034*	
H31B	0.6745	0.6072	0.6479	0.034*	
C32	0.7009 (6)	0.5975 (3)	0.7583 (2)	0.0326 (10)	
H32A	0.7727	0.6602	0.7618	0.049*	
H32B	0.7441	0.5467	0.7933	0.049*	
H32C	0.5764	0.6139	0.7688	0.049*	
C33	0.4142 (5)	0.4697 (3)	0.6858 (2)	0.0221 (9)	
H33A	0.4039	0.4798	0.7388	0.027*	
H33B	0.3724	0.5336	0.6620	0.027*	
C34	0.2925 (6)	0.3820 (2)	0.6635 (3)	0.0313 (10)	
H34A	0.1703	0.3980	0.6780	0.047*	
H34B	0.3309	0.3185	0.6875	0.047*	
H34C	0.2978	0.3730	0.6108	0.047*	

### Atomic displacement parameters (Å^2^)

**Table:**

	*U*^11^	*U*^22^	*U*^33^	*U*^12^	*U*^13^	*U*^23^
O29	0.0224 (15)	0.0231 (12)	0.0244 (16)	0.0027 (12)	−0.0021 (14)	0.0000 (11)
C1	0.021 (2)	0.0181 (16)	0.024 (2)	0.0023 (16)	0.0017 (19)	−0.0008 (16)
C2	0.026 (2)	0.0220 (17)	0.024 (2)	0.0022 (16)	0.000 (2)	0.0014 (16)
C3	0.032 (3)	0.0265 (18)	0.024 (2)	−0.0026 (18)	−0.001 (2)	0.0017 (16)
C4	0.027 (2)	0.0283 (17)	0.019 (2)	−0.0023 (18)	0.001 (2)	−0.0051 (15)
C5	0.026 (2)	0.0229 (16)	0.026 (2)	0.0002 (17)	0.000 (2)	−0.0015 (16)
C6	0.022 (2)	0.0225 (16)	0.025 (2)	0.0038 (16)	0.0009 (19)	−0.0049 (16)
C7	0.027 (2)	0.0220 (17)	0.025 (2)	0.0029 (16)	−0.0023 (19)	−0.0009 (16)
C8	0.019 (2)	0.0231 (16)	0.0194 (19)	−0.0041 (17)	0.0026 (18)	−0.0015 (15)
C9	0.025 (2)	0.0198 (16)	0.021 (2)	−0.0004 (16)	0.0024 (18)	−0.0031 (15)
C10	0.018 (2)	0.0212 (16)	0.022 (2)	0.0003 (16)	−0.0005 (18)	−0.0003 (15)
C11	0.017 (2)	0.0229 (16)	0.019 (2)	−0.0007 (15)	0.0025 (18)	−0.0027 (15)
C12	0.021 (2)	0.0201 (16)	0.022 (2)	−0.0016 (16)	0.0018 (19)	−0.0024 (15)
C13	0.021 (2)	0.0202 (16)	0.022 (2)	−0.0023 (16)	−0.0017 (19)	−0.0011 (15)
C21	0.017 (2)	0.0174 (16)	0.031 (2)	0.0041 (16)	−0.0059 (19)	0.0002 (15)
C22	0.022 (2)	0.0228 (16)	0.026 (2)	0.0021 (17)	0.0021 (19)	0.0003 (16)
C23	0.021 (2)	0.0260 (17)	0.025 (2)	0.0016 (17)	−0.0014 (19)	0.0023 (15)
C24	0.024 (2)	0.0362 (19)	0.027 (2)	−0.0018 (19)	0.004 (2)	−0.0003 (18)
C25	0.027 (2)	0.039 (2)	0.028 (2)	−0.005 (2)	0.000 (2)	0.0063 (18)
C26	0.025 (2)	0.0282 (18)	0.032 (3)	−0.0032 (18)	−0.009 (2)	0.0078 (18)
C27	0.025 (2)	0.0238 (17)	0.027 (2)	−0.0008 (17)	−0.0040 (19)	−0.0010 (17)
C28	0.022 (2)	0.0253 (17)	0.0139 (19)	−0.0060 (17)	−0.0019 (17)	0.0008 (15)
C31	0.033 (3)	0.0232 (17)	0.029 (2)	−0.0009 (18)	0.003 (2)	−0.0008 (16)
C32	0.034 (3)	0.0305 (19)	0.033 (3)	−0.0078 (19)	−0.002 (2)	−0.0069 (17)
C33	0.025 (2)	0.0215 (17)	0.020 (2)	0.0028 (17)	0.0031 (18)	0.0003 (15)
C34	0.027 (2)	0.0326 (19)	0.034 (2)	−0.0024 (19)	−0.001 (2)	0.0010 (18)

### Geometric parameters (Å, °)

**Table:**

O29—C28	1.374 (5)	C12—C13	1.452 (5)
O29—C21	1.395 (4)	C21—C22	1.336 (5)
C1—C10	1.520 (5)	C22—C23	1.438 (5)
C1—C11	1.521 (5)	C22—H22	0.9500
C1—C33	1.539 (5)	C23—C24	1.386 (6)
C1—C31	1.548 (5)	C23—C28	1.397 (5)
C2—C11	1.375 (5)	C24—C25	1.388 (5)
C2—C3	1.389 (5)	C24—H24	0.9500
C2—H2	0.9500	C25—C26	1.400 (5)
C3—C4	1.382 (5)	C25—H25	0.9500
C3—H3	0.9500	C26—C27	1.386 (6)
C4—C5	1.381 (5)	C26—H26	0.9500
C4—H4	0.9500	C27—C28	1.381 (5)
C5—C12	1.391 (5)	C27—H27	0.9500
C5—H5	0.9500	C31—C32	1.512 (6)
C6—C7	1.377 (6)	C31—H31A	0.9900
C6—C13	1.391 (5)	C31—H31B	0.9900
C6—H6	0.9500	C32—H32A	0.9800
C7—C8	1.396 (5)	C32—H32B	0.9800
C7—H7	0.9500	C32—H32C	0.9800
C8—C9	1.410 (5)	C33—C34	1.519 (5)
C8—C21	1.456 (5)	C33—H33A	0.9900
C9—C10	1.374 (5)	C33—H33B	0.9900
C9—H9	0.9500	C34—H34A	0.9800
C10—C13	1.401 (5)	C34—H34B	0.9800
C11—C12	1.412 (5)	C34—H34C	0.9800
			
C28—O29—C21	105.6 (3)	C21—C22—C23	108.0 (3)
C10—C1—C11	101.5 (3)	C21—C22—H22	126.0
C10—C1—C33	112.6 (3)	C23—C22—H22	126.0
C11—C1—C33	112.8 (3)	C24—C23—C28	118.6 (3)
C10—C1—C31	113.0 (3)	C24—C23—C22	136.8 (4)
C11—C1—C31	107.4 (3)	C28—C23—C22	104.6 (3)
C33—C1—C31	109.3 (3)	C23—C24—C25	118.6 (4)
C11—C2—C3	118.7 (3)	C23—C24—H24	120.7
C11—C2—H2	120.6	C25—C24—H24	120.7
C3—C2—H2	120.6	C24—C25—C26	121.2 (4)
C4—C3—C2	121.0 (4)	C24—C25—H25	119.4
C4—C3—H3	119.5	C26—C25—H25	119.4
C2—C3—H3	119.5	C27—C26—C25	121.3 (4)
C5—C4—C3	120.7 (4)	C27—C26—H26	119.4
C5—C4—H4	119.6	C25—C26—H26	119.4
C3—C4—H4	119.6	C28—C27—C26	116.0 (4)
C4—C5—C12	119.0 (3)	C28—C27—H27	122.0
C4—C5—H5	120.5	C26—C27—H27	122.0
C12—C5—H5	120.5	O29—C28—C27	124.9 (3)
C7—C6—C13	119.3 (3)	O29—C28—C23	110.8 (3)
C7—C6—H6	120.3	C27—C28—C23	124.3 (4)
C13—C6—H6	120.3	C32—C31—C1	116.9 (3)
C6—C7—C8	121.2 (3)	C32—C31—H31A	108.1
C6—C7—H7	119.4	C1—C31—H31A	108.1
C8—C7—H7	119.4	C32—C31—H31B	108.1
C7—C8—C9	119.0 (3)	C1—C31—H31B	108.1
C7—C8—C21	120.8 (3)	H31A—C31—H31B	107.3
C9—C8—C21	120.1 (3)	C31—C32—H32A	109.5
C10—C9—C8	120.0 (3)	C31—C32—H32B	109.5
C10—C9—H9	120.0	H32A—C32—H32B	109.5
C8—C9—H9	120.0	C31—C32—H32C	109.5
C9—C10—C13	120.1 (3)	H32A—C32—H32C	109.5
C9—C10—C1	129.4 (3)	H32B—C32—H32C	109.5
C13—C10—C1	110.5 (3)	C34—C33—C1	114.7 (3)
C2—C11—C12	120.6 (4)	C34—C33—H33A	108.6
C2—C11—C1	128.8 (3)	C1—C33—H33A	108.6
C12—C11—C1	110.5 (3)	C34—C33—H33B	108.6
C5—C12—C11	119.8 (3)	C1—C33—H33B	108.6
C5—C12—C13	131.9 (3)	H33A—C33—H33B	107.6
C11—C12—C13	108.3 (3)	C33—C34—H34A	109.5
C6—C13—C10	120.4 (4)	C33—C34—H34B	109.5
C6—C13—C12	130.4 (3)	H34A—C34—H34B	109.5
C10—C13—C12	109.2 (3)	C33—C34—H34C	109.5
C22—C21—O29	110.9 (3)	H34A—C34—H34C	109.5
C22—C21—C8	134.1 (3)	H34B—C34—H34C	109.5
O29—C21—C8	115.0 (3)		
			
C11—C2—C3—C4	−0.9 (6)	C1—C10—C13—C12	−1.4 (4)
C2—C3—C4—C5	0.5 (6)	C5—C12—C13—C6	−2.2 (7)
C3—C4—C5—C12	−0.2 (6)	C11—C12—C13—C6	177.5 (4)
C13—C6—C7—C8	0.9 (6)	C5—C12—C13—C10	179.8 (4)
C6—C7—C8—C9	−1.0 (6)	C11—C12—C13—C10	−0.5 (4)
C6—C7—C8—C21	177.6 (4)	C28—O29—C21—C22	2.2 (4)
C7—C8—C9—C10	0.5 (6)	C28—O29—C21—C8	−175.7 (3)
C21—C8—C9—C10	−178.1 (4)	C7—C8—C21—C22	−171.3 (4)
C8—C9—C10—C13	0.2 (6)	C9—C8—C21—C22	7.3 (7)
C8—C9—C10—C1	179.4 (4)	C7—C8—C21—O29	6.0 (5)
C11—C1—C10—C9	−176.8 (4)	C9—C8—C21—O29	−175.4 (3)
C33—C1—C10—C9	−55.9 (6)	O29—C21—C22—C23	−1.4 (5)
C31—C1—C10—C9	68.5 (5)	C8—C21—C22—C23	175.9 (4)
C11—C1—C10—C13	2.5 (4)	C21—C22—C23—C24	−177.1 (5)
C33—C1—C10—C13	123.3 (3)	C21—C22—C23—C28	0.1 (4)
C31—C1—C10—C13	−112.2 (4)	C28—C23—C24—C25	−0.5 (6)
C3—C2—C11—C12	1.1 (6)	C22—C23—C24—C25	176.5 (4)
C3—C2—C11—C1	177.8 (4)	C23—C24—C25—C26	0.3 (6)
C10—C1—C11—C2	−179.8 (4)	C24—C25—C26—C27	0.1 (6)
C33—C1—C11—C2	59.6 (5)	C25—C26—C27—C28	−0.4 (6)
C31—C1—C11—C2	−61.0 (5)	C21—O29—C28—C27	176.8 (4)
C10—C1—C11—C12	−2.8 (4)	C21—O29—C28—C23	−2.1 (4)
C33—C1—C11—C12	−123.5 (3)	C26—C27—C28—O29	−178.5 (4)
C31—C1—C11—C12	116.0 (3)	C26—C27—C28—C23	0.3 (6)
C4—C5—C12—C11	0.4 (6)	C24—C23—C28—O29	179.1 (4)
C4—C5—C12—C13	−179.9 (4)	C22—C23—C28—O29	1.2 (4)
C2—C11—C12—C5	−0.8 (6)	C24—C23—C28—C27	0.2 (6)
C1—C11—C12—C5	−178.1 (4)	C22—C23—C28—C27	−177.7 (4)
C2—C11—C12—C13	179.4 (4)	C10—C1—C31—C32	−71.1 (5)
C1—C11—C12—C13	2.1 (4)	C11—C1—C31—C32	177.8 (3)
C7—C6—C13—C10	−0.3 (6)	C33—C1—C31—C32	55.1 (5)
C7—C6—C13—C12	−178.1 (4)	C10—C1—C33—C34	−61.3 (5)
C9—C10—C13—C6	−0.3 (6)	C11—C1—C33—C34	52.8 (4)
C1—C10—C13—C6	−179.6 (3)	C31—C1—C33—C34	172.2 (3)
C9—C10—C13—C12	178.0 (4)		

### Hydrogen-bond geometry (Å, °)

**Table:**

Cg1, Cg2, Cg3 and Cg4 are the centroids of the C23–C28, O29/C21–C23/C28 and C2–C5/C11/C12 rings, respectively.

**Table:**

*D*—H···*A*	*D*—H	H···*A*	*D*···*A*	*D*—H···*A*
C2—H2···Cg1^i^	0.95	2.99	3.643 (5)	127
C22—H22···Cg1^ii^	0.95	2.70	3.500 (4)	143
C24—H24···Cg2^ii^	0.95	2.85	3.569 (5)	133
C27—H27···Cg2^iii^	0.95	2.75	3.558 (5)	143

Symmetry codes: (i) −*x*+1, *y*+1/2, −*z*+3/2; (ii) *x*−1/2, −*y*+1/2, −*z*+2; (iii) −*x*+2, *y*−1/2, −*z*+3/2.
